# Hybrid repair of proximal aortic arch pathologies in high-risk surgical patients with extra-anatomic carotid bypass and zone 0 thoracic branched endograft

**DOI:** 10.1016/j.jvscit.2026.102345

**Published:** 2026-06-01

**Authors:** David Parr, Gregory Simonian, David O’Connor, Charles DeCarlo, Anjali Ratnathicam, Kristin Cook, Jinny Lu, Yuriy Dudiy, George Batsides, Mark Anderson, Elie Elmann, Rachel Spallone, Michael Wilderman

**Affiliations:** a Department of Surgery, Hackensack University Medical Center, Hackensack, NJ; b Bergen Vascular Institute, Hackensack, NJ; c Division of Cardiovascular Surgery, Department of Surgery, Hackensack University Medical Center, Hackensack, NJ

**Keywords:** Endovascular surgery, Aortic aneurysm, Zone 0 aortic repair, Gore TAG Thoracic Branched Endoprosthesis

## Abstract

**Objective:**

To demonstrate perioperative outcomes and short-term follow-up from a single-center experience using extra-anatomic carotid bypass and subsequent thoracic endovascular aortic repair using the Gore TAG thoracic branched endoprosthesis for the repair of proximal aortic arch pathologies with a zone 0 landing.

**Methods:**

This retrospective single-center case series reviews consecutive patients with zone 0 aortic pathology treated between October 2023 and December 2025. Patients were determined to be high risk for open intervention by a multidisciplinary board of cardiovascular and vascular surgeons who reviewed each case independently. All patients underwent a staged hybrid approach consisting of extra-anatomic carotid bypass followed by zone 0 thoracic endovascular aortic repair using the Gore TAG thoracic branched endoprosthesis. The primary outcome was technical success, defined as complete exclusion of aortic pathology with patent bypass grafts. Secondary outcomes included 30-day mortality, major adverse events, and early reintervention.

**Results:**

Twenty-two consecutive patients (mean age, 66.7 years; 72.7% male) were treated. Indications included residual type A dissection after earlier open repair (50%), aortic arch aneurysm (27.3%), acute dissection (18.2%), and pseudoaneurysm (4.5%). Technical success was achieved in 100% of cases. Thirty-day mortality was 9.1% (2/22). Major adverse events included stroke in one patient (4.5%) and respiratory failure in two patients (9.1%). Dysphagia occurred in 27.3% and was self-limited in most cases. Early endoleaks were identified in 22.7%, with one requiring reintervention.

**Conclusions:**

A staged hybrid approach using extra-anatomic bypass and zone 0 thoracic branched endograft repair is feasible and reproducible in high-risk patients, with favorable short-term outcomes and acceptable morbidity.

**Article Highlights**

• **Type of Research:** Single-center retrospective case series
• **Key Findings:** Staged hybrid repair of zone 0 aortic pathology by extra-anatomic carotid bypass and subsequent zone 0 Gore TAG thoracic branched endograft in 22 patients was found to be technically feasible, with 100% intraoperative success and comparable 30-day mortality (9.1%, n = 2) relative to reported outcomes of open or hybrid repair options.
• **Take Home Message:** Staged hybrid repair of zone 0 lesions using extra-anatomic carotid bypass and Gore thoracic branched endoprosthesis appears to be a safe and effective treatment option for patients determined to be high risk for open surgical intervention.

Aortic pathology remains a major cause of morbidity and mortality in the United States. Thoracic endovascular aortic repair (TEVAR) has become the standard treatment for most distal arch and descending aortic pathologies.^1^ However, for ascending aortic and proximal arch disease, open repair remains the gold standard despite significant risks of morbidity, mortality, and prolonged hospitalization. Endovascular treatment options for the ascending aorta and proximal arch remain limited, with current clinical investigation still in its early stages.

In 2022, the Gore TAG thoracic branched endoprosthesis (TBE; W.L. Gore & Associates) was approved for the treatment of lesions in aortic zone 2, enabling reliable management of pathology at the level of the left subclavian artery (LSA).^2^ This device has been rapidly incorporated into clinical practice and has demonstrated consistent technical success with low complication rates across a range of aortic pathologies, including aneurysm, acute dissection, and traumatic injury.^3^^,^^4^ Despite such advances in the descending aorta and distal arch, endovascular management of ascending aortic pathology continues to pose substantial challenges.

Ascending aortic pathology is diverse, including aneurysm, dissection, pseudoaneurysm, and a number of less common lesions. Type A aortic dissection accounts for approximately 60% of all thoracic aortic dissections.^5^^,^^6^ Although progress has been made in recent decades, open surgical repair continues to carry considerable risk, with reported mortality ranging from 17% to 26% in the International Registry for Acute Aortic Dissection database^7^ and a 30-day mortality of 10.4% in a recent meta-analysis.^8^ Over the past two decades, various hybrid approaches have been described for complex aortic disease involving zones 0 and 1, particularly in acute dissection. These hybrid approaches typically combine open repair of the ascending aorta and arch debranching with subsequent endovascular stent graft deployment into the replaced ascending aorta. However, open sternotomy is often still required, and hybrid approaches have not demonstrated improved 30-day mortality compared with conventional open repair.^8^

In June 2025, the Gore TAG TBE received approval for use in lesions involving aortic zones 0 and 1, expanding endovascular options for proximal aortic arch repair. Although this development offers new opportunities for minimally invasive treatment, use of the device remains limited by aortic arch anatomy and available landing zones.

Here, we describe a single-center case series of reproducible hybrid repair for patients with zone 0 aortic disease who were determined to be ineligible for open surgical repair or required reintervention for residual or recurrent dissection following previous open repair. These patients were managed using a minimally invasive staged hybrid approach consisting of extra-anatomic carotid bypass followed by zone 0 TEVAR with the Gore TBE device, constituting one of the first reported series following the recent Food and Drug Administration approval. Many of the patients treated in the presented series underwent intervention before Food and Drug Administration approval of this device for the treatment of zone 0 lesions. Each patient understood that the device being used was commercially available and frequently used in aortic interventions but was being used outside its specific design to treat their unique aortic pathology.

## Methods

This case series reports the clinical efficacy of a staged hybrid approach for managing complex aortic pathology involving the ascending aorta and proximal arch. Ethics committee approval was acquired for chart review. TeraRecon and 3mensio were both used for surgical planning, and all cases were reviewed by a team of vascular and cardiac surgeons, as well as representatives from W.L. Gore & Associates. The treatment algorithm consisted of an extra-anatomic carotid bypass followed by zone 0 TEVAR using the Gore TBE device. Consecutive patients treated with this protocol between October 2023 and December 2025 were included (Table I). Data were collected via retrospective chart review, assessing technical success, overall mortality, relevant complications, and freedom from reintervention.

**Table I:** Patient demographics and procedural specifics

Patient demographics (N = 22)	
Age, years	66.7 ± 12.9
Sex, male	16 (72.7)
Length of stay	7.5 ± 5.3
Indication	
Residual TAAD after previous open repair	11 (50)
Aortic arch aneurysm	6 (27.3)
Aortic arch dissection	4 (18.2)
Aortic arch pseudoaneurysm	1 (4.5)
Length of bypass	
Right common carotid to left subclavian	18 (81.8)
Right common carotid to left common carotid	3 (13.6)
Left common carotid to left subclavian	1 (4.5)
Endovascular landing zone	
Zone 0	22 (100)

*TAAD*, Type A aortic dissection.

Continuous data are presented as mean ± standard deviation (range), and categorical variables are presented as count, expressed as “No. (%).”

All patients underwent extra-anatomic carotid bypass, with most patients requiring a right common carotid artery (RCCA) to LSA bypass using an 8-mm Dacron graft tunneled retropharyngeally, with proximal left common carotid artery (LCCA) ligation and reimplantation of the distal LCCA onto the LSA bypass graft (81.8%, n = 18). Three patients required only RCCA to LCCA bypass (13.6%), as they each had an existing LCCA to LSA bypass from earlier procedure. One patient underwent partial arch debranching during a previous procedure and only required an LCCA to LSA bypass (4.5%).

Using a staged approach, all patients underwent subsequent TEVAR using the Gore TBE device deployed in zone 0 with branch placement in the innominate artery, with proximal or distal extension as needed to acquire adequate seal. The Gore Conformable TAG (C-TAG) device was frequently used to extend the proximal extent of aortic coverage to achieve adequate seal. This device allowed for conformation of the endograft across the aortic arch in cases with pathology extending proximal to the innominate branch of the TBE device or in cases where the intended landing zone was a short or angulated surgical graft from prior open surgical repair. In all cases, standard Gore TBE grafts (34-40 mm main body endografts, 15-17 mm branch components) were used in the innominate artery. One patient was found to have dissection extension into the RCCA, requiring placement of a bare metal stent across the origin of the right subclavian artery—a Zilver VENA stent (Cook Medical) was used given its diameter and open-cell structure. Completion angiography confirmed perfusion of the cerebral and upper extremity circulation through the innominate branch and carotid bypass graft (Fig). The endovascular procedure occurred within 24 hours for all but one patient (95.5%), with one patient requiring a 3-day interval between procedures. All patients who underwent carotid bypass ultimately proceeded to endovascular repair, and no patient suffered complications during the first procedure which excluded them from TEVAR.

**Fig:**
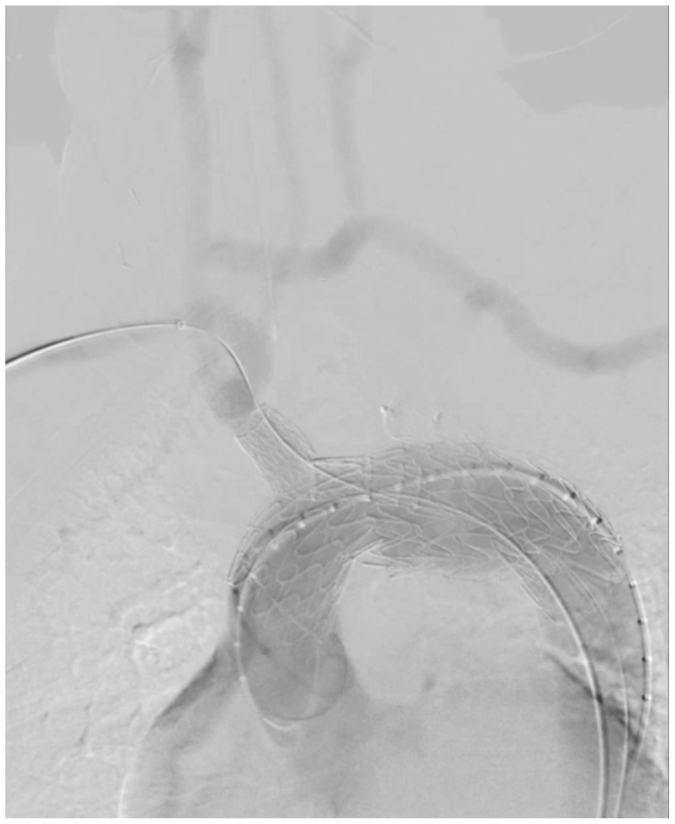
Successful deployment of Gore TAG thoracic branched endograft with branch into the innominate artery supplying the carotid arteries and left subclavian artery via a patent carotid-carotid-subclavian bypass graft.

Image used with expressed patient consent.

The primary outcome was technical success, defined as complete exclusion of the aortic pathology with patent bypass grafts. Secondary outcomes included 30-day mortality, length of stay, and major adverse events from either procedure, including myocardial infarction, stroke, respiratory failure, visceral malperfusion, renal failure, paralysis, nerve injury, and dysphagia. Postoperative surveillance with computed tomography (CT) imaging assessed bypass patency, presence of endoleaks, and freedom from reintervention.

## Results

Over a 26-month period, 22 patients with proximal aortic pathology deemed unfit for open surgical repair underwent a staged hybrid endovascular approach at a tertiary care hospital with a dedicated multidisciplinary aortic center. Patient age ranged from 46 to 93 years (mean 66.7 years), with a male predominance (72.7%, 16/22). The most common indication for intervention was residual type A dissection following previous open repair (n = 11), followed by aortic arch aneurysm (n = 6), initial type A or arch dissection (n = 4), and pseudoaneurysm (n = 1). Mean hospital length of stay was 7.5 days (range, 3-24 days).

Technical success was achieved in all cases, defined as patent bypass grafts (RCCA-LCCA-LSA or LCCA-LSA), complete exclusion of the aortic pathology, and absence of endoleak on completion imaging. Thirty-day mortality was 9.1% (2/22) (Table II). The first death occurred in a 93-year-old patient who suffered a perioperative stroke complicated by acute hypoxic respiratory failure and was transitioned to comfort measures on postoperative day 11. The second occurred in an 82-year-old patient who developed postoperative atrial fibrillation and progressive cardiogenic shock in addition to acute hypoxic respiratory failure and was also transitioned to comfort measures on postoperative day 10.

**Table II:** Postoperative outcomes within 30 days

Postoperative outcomes	
Primary outcome, technical success	
Exclusion of aortic pathology	22 (100)
Patent bypass graft	22 (100)
Major adverse events	
30-day mortality	2 (9.1)
Major adverse cardiac event	0
Acute hypoxic respiratory failure	2 (9.1)
Cerebrovascular event or stroke	1 (4.5)
Visceral malperfusion	0
Renal injury or failure	0
Neurological complication (paralysis and nerve injury)	0
Surgical complications	
Local wound complications	1 (4.5)
Endoleak: type 1A and type 2	4 (18.2)
Endoleak requiring intervention: type 3	1 (4.5)
Respiratory complications	
Respiratory failure requiring prolonged intubation	2 (9.1)
Readmission for respiratory distress	2 (9.1)
Discharged on home O_2_	1 (4.5)
Cardiac complication	
New atrial fibrillation	1 (4.5)
GI complication	
Mild- or self-limited dysphagia	5 (22.7)
Dysphagia requiring intervention	1 (4.5)
Hospital readmission within 30 days	2 (4.5)

*GI*, Gastrointestinal.

Categorical variables are presented as count, expressed as “No. (%)”.

Major adverse events included respiratory failure in the two patients as discussed previously, both requiring prolonged intubation before transitioning to end-of-life care (9.1%). Two additional patients with pre-existing pulmonary disease were readmitted within 30 days for chronic obstructive pulmonary disease exacerbation (9.1%), and one patient with pre-existing pulmonary disease required home oxygen therapy at discharge (4.5%). None of the respiratory complications were directly attributed to technical complications of either the bypass procedure or the subsequent TEVAR. Dysphagia was the most common postoperative complication, occurring in six patients (27.3%); five cases spontaneously resolved and the sixth patient underwent laryngoscopy, but the dysphagia ultimately resolved without further intervention. One right-sided neck seroma required operative drainage within 30 days (4.5%).

Follow-up imaging was available for 15 patients (75.0%) at a mean of 66.8 days (range, 1-222 days); 2 patients have not yet reached the interval for follow-up imaging. Per study protocol, routine follow-up imaging was performed at 1 month postprocedure, with repeat imaging at 6 or 12 months based on evaluation of the initial imaging. Select high-risk patients underwent CT angiography before discharge, with repeat imaging at 1 or 6 months based on evaluation of the initial imaging. Despite universal intraoperative technical success, five patients (22.7%) demonstrated postoperative endoleaks on follow-up imaging (one type 1A, three type 2, and one type 3); only the type 3 endoleak required reintervention. The identified endoleaks have not undergone angiography for further characterization, though evaluation of CT imaging suggests that all three type 2 endoleaks originate from intercostal arteries. No cases of bleeding, hematoma, neurological injury, spinal cord ischemia, or paralysis were observed. Overall, three patients (13.6%) experienced Clavien-Dindo class III complications, no patients experienced class IV events, and two (9.1%) experienced a class V outcome.

## Discussion

This single-center experience contributes to the growing body of literature supporting endovascular intervention in the ascending aorta by adding real-world data that validate the use of this technique in the management of a broad set of clinical applications. The presented data suggest that extra-anatomic bypass combined with endovascular repair can provide a feasible alternative to open repair in selected patients. Although this retrospective review lacks a direct comparative analysis to the gold standard of open surgical repair, such assessment is inherently challenging, as this technique is primarily applied in patients unsuitable for open repair or those who have undergone earlier open repair. This management approach has the potential to shift the treatment paradigm for this complex and comorbid patient population.

To date, the available literature exists to support the use of the TBE device in zone 0 and zone 1, with detailed inclusion and exclusion criteria across a number of high-volume academic centers.^9^ This review expands on that data by including consecutive patients evaluated at a single high-volume center, with diverse indications reflective of everyday clinical practice. The patient selection in this review was kept intentionally broad to represent real-world use of this technique. Each patient was not eligible for open surgical intervention as determined by the cardiothoracic surgery team, and despite thorough discussion regarding the goals and risks of the procedure, these patients and their families opted to proceed with all possible interventions.

The anatomy of the aortic arch vessels remains the primary limitation to endovascular repair of the ascending aorta and arch. Most manufacturer guidelines recommend a minimum 2 cm landing zone to achieve an adequate seal, which demands extensive preoperative planning for lesions requiring device placement in zones 0 or 1 to avoid cerebrovascular complications, which is precluded in emergent cases. Several strategies to address these complex landing zones have been described since the early 2000s, including transostial bare metal stents, transposition of arch vessels, open bypass grafting, and intentional coverage or occlusion of arch vessels.^10^ Total arch debranching followed by TEVAR has since been demonstrated as a technically feasible option, with a recent 27-patient series reporting a 30-day mortality of 11.1% and a perioperative morbidity rate of 90% when zone 0 TEVAR was performed.^11^ The data presented in this report include a 30-day mortality rate of 9.1%, comparing favorably with previously reported mortality for open or hybrid zone 0 repairs, which typically range from 10% to 25%.^7^, ^8^, ^9^, ^10^, ^11^

Compared with alternative techniques for repair of proximal aortic lesions, the presented data included two patient mortalities (9.1%). Both mortalities occurred in elderly patients with reasonable baseline function who underwent technically successful surgical intervention but ultimately suffered complications related to their existing comorbidities in the postoperative period and transitioned to comfort measures. One of these patients suffered a postoperative stroke, which hastened her transition to comfort measures—while her cerebrovascular accident may have occurred as a result of either procedure, the patient was neurologically intact for 3 days following her TEVAR, during which her anticoagulation for atrial fibrillation had not been resumed, leaving some ambiguity as to the etiology of her embolic stroke. Although these two patients represent a small percentage of the total cohort, discussions with patients and families regarding surgical management in the elderly are understandably nuanced and remain an area for improvement across the fields of cardiothoracic and vascular surgery.

Multiple endovascular techniques have also been developed for the management of aortic arch disease, including physician-modified endografts, in situ laser fenestration, chimney grafts, and multibranched or total endovascular aortic debranching. These approaches have been reported in small series with variable success.^12^, ^13^, ^14^, ^15^ Several ascending aortic endografts have been developed but are not currently commercially available in the United States.^16^

The diversity of techniques for proximal aortic repair reflects the absence of a single superior approach. The complexity of these lesions, combined with the technical challenges of advanced endovascular methods, underscores the need for reproducible strategies. The described approach in this report, consisting of extra-anatomic bypass followed by deployment of a zone 0 Gore TBE, offers a consistent and technically reproducible alternative. It avoids entry into the thoracic cavity, uses familiar endovascular principles, and mirrors standard zone 2 deployment of a commercially available device. This approach may also be more accessible to vascular surgeons than more technically challenging options such as in situ fenestration or physician-modified devices. The ready availability of the Gore TBE device also enables its use as an off-the-shelf option in urgent or emergent settings.

Occult endoleaks remain a potential pitfall of all endovascular aortic interventions. In the reported dataset, the authors defined technical success as the absence of identifiable endoleak on completion angiography, with intentionally close follow-up and routine imaging at 1 month. Of the five endoleaks identified on follow-up imaging, only one required reintervention. A type 1A endoleak was identified on follow-up imaging at 7 days, before discharge home from the hospital. The patient had anomalous anatomy (truncus bicaroticus), and the initial intervention was performed for a complex residual type A dissection following earlier open repair. The endoleak was identified on delayed images on the first CT angiography, and contrast filling did not propagate into the arch or proximally toward the coronary arteries, so the decision was made to defer further intervention. Ultimately, the endoleak nearly resolved on subsequent CT imaging.

Although this report highlights the success of a single-center's application of the Gore TBE device in a staged hybrid technique, randomized controlled trials are vital to delineate generalizable patient outcomes. In addition, as with many modern endovascular interventions, the long-term outcome data remain limited. Ongoing follow-up is crucial to characterize the impact of proximal endograft placement, particularly given the frequency of type A aortic dissection in relatively young patients. The long-term effects of endograft placement within the ascending aorta and across the aortic arch are not yet well understood, emphasizing the need for continued surveillance and imaging beyond early postoperative assessments. Continued efforts are also needed to better define characteristics that preclude patients from conventional open surgery, as clearer selection criteria will be essential for broader evaluation and adoption.^17^

A gap persists in the management of aortic pathology. TEVAR and the Gore TBE device have proven effective for distal arch and descending thoracic disease, and open repair remains appropriate for selected proximal lesions. As the global patient population ages and becomes more comorbid, the proportion of patients unable to tolerate open repair will continue to increase. The need for minimally invasive and hybrid options for proximal aortic repair in these patients will continue to expand. The staged hybrid technique presented here supports hybrid repair as technically feasible, reproducible, and associated with short-term outcomes comparable to those reported in existing literature.

## Conclusions

This retrospective review of a single-center case series demonstrates the feasibility of staged hybrid repair of ascending aortic and proximal aortic arch pathologies using the Gore TBE device in zone 0. The technique leverages familiar open and endovascular principles and provides excellent technical outcomes and favorable short-term results. As the global patient population ages and becomes increasingly comorbid, hybrid repair options may expand the role of endovascular intervention in the management of proximal aortic lesions. Further clinical investigation in larger prospective cohorts should address the durability of repair and long-term outcomes in these patients with no clear surgical alternative.

## Author Contributions

Conception and design: DP, GS, DO, CD, AR, KC, JL, YD, GB, MA, EE, RS, MW

Analysis and interpretation: DP, GS, DO, MW

Data collection: DP, GS, DO, CD, AR, KC, JL, MW

Writing the article: DP, GS, MW

Critical revision of the article: DP, GS, DO, CD, AR, KC, JL, YD, GB, MA, EE, RS, MW

Final approval of the article: DP, GS, DO, CD, AR, KC, JL, YD, GB, MA, EE, RS, MW

Statistical analysis: Not applicable

Obtained funding: Not applicable

Overall responsibility: MW

## Funding

None.

## Disclosures

None.
